# Access to Vaccination for Newly Arrived Migrants: Developing a General Conceptual Framework for Understanding How to Improve Vaccination Coverage in European Countries

**DOI:** 10.3389/ijph.2023.1605580

**Published:** 2023-08-07

**Authors:** Salvatore Scarso, Giulia Marchetti, Maria Laura Russo, Franca D’Angelo, Maria Elena Tosti, Arianna Bellini, Chiara De Marchi, Caterina Ferrari, Angela Gatta, Susanna Caminada, Nikoletta Papaevgeniou, Nadia Dalma, Pania Karnaki, Maurizio Marceca, Silvia Declich

**Affiliations:** ^1^ National Center for Global Health, National Institute of Health (ISS), Rome, Italy; ^2^ Department of Public Health and Infectious Diseases, Sapienza University of Rome, Roma, Italy; ^3^ Italian Society of Migration Medicine (SIMM), Rome, Italy; ^4^ Prolepsis Institute for Preventive Medicine and Environmental and Occupational Health, Marousi, Greece

**Keywords:** vaccination coverage, newly arrived migrants, general conceptual framework, system barriers, strategies

## Abstract

**Objectives:** Access to vaccination for newly arrived migrants (NAMs) is a relevant concern that requires urgent attention in EU/EEA countries. This study aimed to develop a General Conceptual Framework (GCF) for understanding how to improve vaccination coverage for NAMs, by characterizing and critically analyzing system barriers and possible strategies to increase vaccination.

**Methods:** A theoretical conceptualization of the GCF was hypothesized based on conceptual hubs in the immunization process. Barriers and solutions were identified through a non-systematic desktop literature review and qualitative research. The GCF guided the activities and facilitated the integration of results, thereby enriching the GCF with content.

**Results:** The study explores the vaccination of NAMs and proposes strategies to overcome barriers in their vaccination process. It introduces a framework called GCF, which consists of five interconnected steps: entitlement, reachability, adherence, achievement, and evaluation of vaccination. The study also presents barriers and solutions identified through literature review and qualitative research, along with strategies to enhance professionals’ knowledge, improve reachability, promote adherence, achieve vaccination coverage, and evaluate interventions. The study concludes by recommending strategies such as proximity, provider training, a migrant-sensitive approach, and data collection to improve vaccination outcomes for NAMs.

**Conclusion:** Ensuring equitable access to healthcare services, including vaccination, is crucial not only from a humanitarian perspective but also for the overall public health of these countries.

## Introduction

Access to immunization for migrants, especially for newly arrived migrants (NAMs) is a critical aspect of public health interventions in the European Union and Economic European Area (EU/EEA) [1]. Immunization is a key global health intervention that saves millions of lives and prevents the spread of numerous life-threatening diseases [1].

Migrants may not be immunized or may be under immunized in their countries of origin and so may be vulnerable to acquire Vaccine Preventable Diseases (VPD). Scientific evidence strongly supports the importance of vaccinations for protecting migrant populations from infectious diseases. Migrants are at higher risk due to unsanitary conditions and limited access to healthcare. Vaccinations are an effective way to prevent the spread of diseases among migrant populations, ensuring their health and the health of the communities they join. Access to immunization services for migrants is crucial for preventing disease outbreaks and promoting their wellbeing and integration into society [2–5]. In the WHO European region, migrants and refugee children constitute about 25% of the total migrant population, making them one of the groups most at risk for vaccine-preventable diseases [6].

In 2019, the WHO published a technical guide outlining three essential elements to ensure high vaccination coverage among refugees and migrants: appropriate vaccination services for newly arrived individuals, delivery of immunization services as part of mainstream health services, and targeted and culturally appropriate immunization services [4]. Moreover, in 2020, all WHO member states endorsed the Immunization Agenda 2030: A Global Strategy to Leave No One Behind (IA2030) [7]. This agenda is organized into seven strategic priorities for immunization in the next decade and is guided by four core principles: people-centered, country-owned, partnership, and data-guided. National and international public health institutions have developed strategies to address the immunization of migrants in their national health systems, despite these efforts, challenges remain in ensuring optimal vaccination coverage for this vulnerable population [2–5]. To further address this issue, the EU has also launched funding opportunities such as the Access to Vaccination for Newly Arrived Migrants (AcToVax4NAM) project, which intends to improve vaccination literacy (VL) and access and thereby vaccination coverage for NAMs in first-line and destination countries making access conditions more equitable and guaranteed [8].

This study aims to develop a General Conceptual Framework (GCF) to understand how to improve vaccination coverage among migrants in EU/EEA countries. Considering all steps taken in the healthcare pathway from the vaccination entitlement to completion of needed vaccination, addressing also dropout, and adopting a life‐course approach to immunization for children, adolescents, adults and elderly. By reviewing existing policies, strategies, and initiatives, including those from the WHO and EU-funded projects, the GCF identifies system barriers and best strategies as well as potential areas for improvement to ensure equitable access to vaccination for all, including NAMs. In doing so, we hope to contribute to the ongoing efforts to achieve universal health coverage and protect public health across EU countries.

## Methods

The GCF was developed through three steps, using mixed-methods: theoretical conceptualization of a Preliminary Conceptual Framework (PCF), non-systematic desktop review and qualitative research (focus groups and personal interviews) and consolidation of the GCF.

### Theoretical Conceptualization of a PCF—Step 1

As first step, the extended expertise on migrant health and immunization systems of the National Center for Global Health, Istituto Superiore di Sanità and the Department of Public Health and Infectious Diseases, Sapienza University of Rome staff was used as key resource to conceptualize, identify and design the logical process of an inclusive immunization program for NAMs. The development of the PCF started with a hub-and-spoke model during a hybrid (in-person and virtual) workshop. Furthermore, to be able to frame the different issues and understand the reasoning behind each hub, the team has formulated specific “Question Groups” (QG) concerning every hub. All questions refer to NAMs according to the AcToVax4NAM operational definition “*A person (with a different citizenship from the hosting country, with either EU/EEA or third country citizenship), who entered the country in the last 12 months EITHER within the procedures prescribed by the governmental migration policies, excluding tourists and short visa/permit <3 months, OR outside the procedures recognized by the legislation (or overstay after visa expired)*” and for all questions, distinctions between different legal status of NAMs should be considered. These questions were a useful tool to accurately guide personal interviews/focus groups and characterize and assign all records extracted from the literature review and the qualitative research in the corresponding hub (see Supplementary Material S1).

### Non-Systematic Desktop Review and Qualitative Research—Step 2

#### Non-Systematic Desktop Review

A desktop literature review has been implemented, also at country level, to identify existing research concerning system barriers (at legal, economic, organizational, psycho-social and cultural-linguistic level) and solutions, recommended or suggested, to overcome them. A search strategy was launched on PubMed in order to find scientific articles or documents concerning the topic of interest. The consortium countries integrated the search with materials in local language or contained in national websites (Scientific literature, Guidance, Guideline, Bulletin, Report, Legislation, Policy document, Standard operating procedure). The Supplementary Material contains full details of the search strategy including inclusion and exclusion criteria (see Supplementary Material S2).

#### Qualitative Research (Focus Groups and Personal Interviews)

Focus groups (FGs) and Personal Interviews (PIs) were conducted in Germany, Poland, Spain, Italy, Greece, Malta, and Cyprus, partners of AcToVax4NAM consortium, in order to understand the actual experiences of the “professionals FOR health” (all professionals who must/can deal with the health of migrants) involved in NAMs immunization and to achieve the characterization of system barriers (legal, economic, organizational, psycho-social and cultural-linguistic) and identification of possible and sustainable solutions at country level. Eligible participants included: 1) health and social care professionals who work in the field of delivery of immunizations, 2) professionals, who work in managing/organizing immunization services, and 3) experts related to immunization planning. Guidelines for performing FGs discussion and PIs were developed for all partners by Prolepsis Institute to provide a common methodology and ensure a uniform group composition (see Supplementary Material S3). FGs discussions and PIs were transcribed verbatim in local languages and identifiers were removed to maintain the anonymity of participants. Transcripts were analyzed using thematic analysis [9].

### Integration of Results and Consolidation of the GCF—Step 3

The GCF and the QGs were utilized to determine and merge the barriers and solutions found during the reading of the desktop literature review and the thematic analysis of the qualitative research. This selection and combination process was guided by their similarities. Subsequently the partners, especially the members of the AcToVax4NAM Steering Committee whichcomprises the project manager and the 6 WP leaders and is the key decision-making and issue-resolution body for the project, sent a two-round of suggestions and comments according to the AcToVax4NAM evaluation plan. These were used for a further revision of the findings, which was then presented to the project Steering Committee meeting and finalized by the end of June 2022.

## Results

**FIGURE 1:**
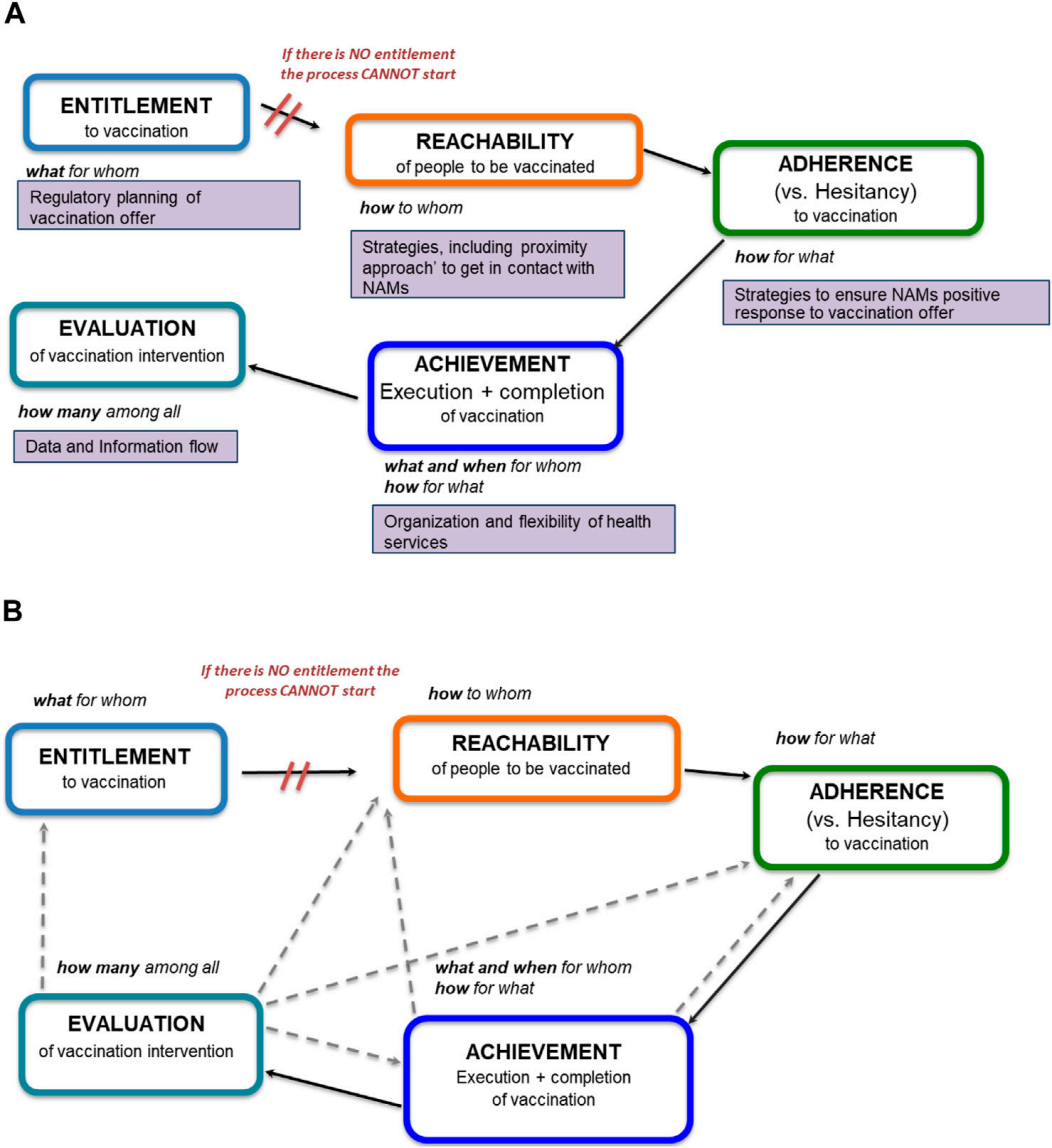
Theoretical conceptualization of a preliminary conceptual framework **(A, B)**, EU/EEA, 2022.

The logical conceptualization considered the main steps involved in the vaccination of NAMs. These steps become the five hubs of the Preliminary Conceptual Framework: 1) *Entitlement* to vaccination, 2) *Reachability* of people to be vaccinated, 3) *Adherence* (vs. Hesitancy) to vaccination, 4) *Achievement* of vaccination (execution and completion), 5) *Evaluation* of vaccination intervention (Figures 1A, B). As can be seen, there is a connection between each hub. The interrupted arrow starting from the *Entitlement* hub underlines that without the legal right to immunisation the entire process cannot start. The continuous arrows show the sequential continuity of the process.

*Entitlement* intended to encapsulate “what for whom” and concerns the regulatory planning of the vaccination offer; *Reachability* is related to “how to whom” and regards all strategies, including the “proximity approach,” and abilities of the health service to get in contact with NAMs; *Adherence* that framed “how for what” includes the strategies to ensure that NAMs respond positively to the vaccination offer and to devise abilities in the “professional FOR health” to counteract vaccination hesitancy and fear among NAMs; *Achievement* concerns the execution and completion of vaccination, that is “what (vaccines) and when for whom and how for what” and should depends on organization and flexibility of health services; *Evaluation*, which should report “how many (vaccinated) among all migrants,” regards the necessity of data collection and information flow about NAMs vaccination to be used for the evaluation of activities (Figure 1A).

The dashed arrows in Figure 1B underline that the *Achievement* and *Evaluation* are linked with the other hubs. In particular, the dashed arrows starting from the *Achievement* hub indicate that if the execution and completion of vaccination do not happen it is important to go back to the previous hubs (*Reachability* and *Adherence*) to understand the reasons. The dashed arrows from the Evaluation hub indicate that the evaluation process must be cross-cutting at all hubs and has to take into account their strategies and actions.

### Barriers and Solutions From Literature Review

One-hundred and fifty-one documents were collected from the review, 85 documents (out of which 38 scientific articles, 16 reports, 5 guidelines, 4 policy documents, 5 technical documents, and 17 other document types), containing at least one barrier and/or solution, were selected and analyzed. After the exclusion of 7 documents (not pertinent to immunization), 78 documents were included [10–20], [21–26], [5,27–33], [2,34–40], [41–79], [4,80–83]. From them, 403 records were extracted, containing barriers (210 records) and possible solutions (193 records). The table in Supplementary Material S3 reports a list of references where at least a barrier and/or a solution related to specific hub can be found.

**FIGURE 2:**
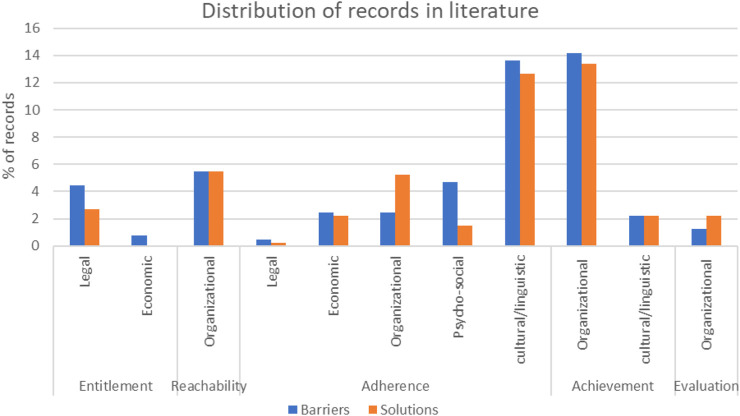
Distribution of barriers and solutions subcategory in literature, EU/EEA, 2022.

By using the QGs, each record was characterized in barriers (52.1%) and solutions (47.1%) and then assigned to related sub-categories. A total number of 32 records were assigned to *Entitlement*, including legal barriers (4.5%) and solutions (2.7%), economic barriers (2.7%) while no economic solutions were found; 44 records were assigned to *Reachability*, including organizational barriers (5.5%) and solutions (5.5%); 184 records were assigned to *Adherence*, including legal barriers (0.5%) and solutions (0.2%), economic barriers (2.5%) and solutions (2.2%), organizational barriers (2.5%) and solutions (5.2%), psycho-social barriers (4.7%) and solutions (1.5%) and cultural/linguistic barriers (13.6%) and solutions (12.7%); 129 records were assigned to *Achievement*, including organizational barriers (14.1%) and solutions (13.4%), cultural/linguistic barriers (2.2%) and solutions (2.2%) and 14 records to *Evaluation*, including organisational barriers (1.2%) and solutions (2.2%) (Figure 2).

### Qualitative Research Findings: Descriptive Characteristics of Participants

In total, 117 people participated in 13 FGs and 53 PIs in Germany, Poland, Spain, Italy, Greece, Malta, and Cyprus. Demographic characteristics of health and social care professionals are showed in Table 1.

**TABLE 1:** Demographic characteristics of health and social care professionals who work in the field of delivery of immunizations (Focus Group 1—FG1), health and social care professionals working in the management/organization of immunization services for minors and/or adult migrants. (Focus Group 2—FG2) and experts related to immunization planning (Personal Interviews—Pis.), EU/EEA, 2022.

	FG1 (*n* = 30)	FG2 (*n* = 35)	Pis (*n* = 52)
Age (years)	45.6 ± 12.7	42.4 ± 9.1	37.2 ± 13.1
Sex (females)	22 (73.3%)	22 (62.9%)	33 (63.5%)
Education (FG1 = 23, FG2 = 23, Pis = 48)			
Upper secondary education	1 (4.4%)	n/a	n/a
Post-secondary non-tertiary	2 (8.7%)	n/a	25 (52.1%)
Tertiary	10 (43.5%)	8 (26.7%)	4 (8.3%)
Master	10 (43.5%)	19 (63.3%)	15 (31.3%)
Doctoral	n/a	3 (10%)	4 (8.3%)
Occupation			
Physician	13 (43.3%)	8 (22.9%)	6 (11.5%)
Manager	n/a	2 (5.7%)	1 (1.9%)
Nurse	10 (33.3%)	2 (5.7%)	8 (15.4%)
Policymaker	n/a	3 (8.6%)	n/a
Administrative staff	2 (6.7%)	3 (8.6%)	25 (48.1%)
Psychologist	n/a	2 (5.7%)	2 (3.8%)
Social worker	2 (6.7%)	4 (11.4%)	1 (1.9%)
Cultural mediator	2 (6.7%)	1 (2.9%)	n/a
Expert	1 (3.3%)	n/a	4 (7.7%)
Other	n/a	10 (28.6%)^a^	5 9.6%)^b^
Institution/organization			
Health center	9 (30%)	7 (20%)	9 (17.3%)
Vaccination local/national units	3 (10%)	2 (5.7%)	3 (5.8%)
NGOs	5 (16.7%)	12 (34.3%)	7 (13.5%)
Entry camps	n/a	2 (5.7%)	n/a
First reception	n/a	1 (2.9%)	n/a
Detention	1 (3.3%)	n/a	n/a
State level organization	2 (6.7%)	6 (17.1%)	n/a
Municipality	4 (13.3%)	4 (11.4%)	n/a
Hospital	n/a	1 (2.9%)	16 (30.8%)
University	1 (3.3%)	3 (8.6%)	13 (25%)
Regional health service	1 (3.3%)	n/a	2 (3.8%)
Refugee camp	1 (3.3%)	4 (11.4%)	2 (3.8%)
Other	9 (30%)^c^	6 (17.1%)^d^	4 (7.7%)^e^
Concerning immunization do you work mainly with			
Children	13 (43.3%)	17 (50%)	30 (57.7%)
Adolescents	19 (63.3%)	24 (70.6%)	29 (55.8%)
Adults	23 (76.7%)	26 (76.5%)	45 (86.5%)
Elders	8 (26.7%)	12 (35.3%)	30 (57.7%)
Concerning newly arrived migrants (NAMs), do you work mainly with (FG1 = 16 FG2 = 25 Pis = 46)			
I don’t work with NAMs	3 (18.8%)	5 (20%)	2 (4.3%)
Documented NAMs	14 (87.5%)	17 (68%)	43 (93.5%)
Undocumented NAMs	12 (75%)	17 (68%)	18 (39.1%)
Resident migrants	8 (50%)	15 (60%)	16 (34.8%)
Refugees	1 (6.3%)	2 (8%)	6.3 ± 7.5
Years working in the area of immunization	10.9 ± 10.1	7.1 ± 7.3	2 (4.3%)

^a^ Including volunteer, legal advisor, project assistant, coordinator, migrant community leader, researcher and public health professional.

^b^ Including IT officer, ex minister of health, health professional working at United Nations High Commissioner for Refugees (UNHCR), community health agent, CEO, Specialist institution for legal care work.

^c^ Including local health unit, scientist, researcher, Hellenic Red Cross, AWO SH.

^d^ Including United Nations High Commissioner for Refugees (UNHCR) and Hellenic Red Cross, MiMi Hamburg.

^e^ Including Public Health National Unit—Infectious Disease Prevention and Control Unit, Regional Level Organization—Department of Prevention and UN High Commissioner for refugees.

### Integration of Results and Consolidation of the GCF

The methodology that involved the integration of different research tools (desktop literature review, focus group, personal interview) into the Preliminary Conceptual Framework permitted a deepening of the processes related to the theoretical conceptual schema produced. The integrated analysis of the findings produced a “fill-in” of the conceptual hubs, previously identified in a theoretical way, producing a greater amplitude, depth and complexity of the dynamics that link the logical hubs of the NAMs vaccination process. In particular, there was a focus on the barriers and solutions that both the literature and the qualitative research identified as relevant in the vaccination process with the aim of making the research more linked to day-to-day operations and stressing their problems and deficiencies. Key findings from Desktop Literature Review and Qualitative research (FGs discussions and Pis) are summarized in Supplementary Material S4.

**FIGURE 3:**
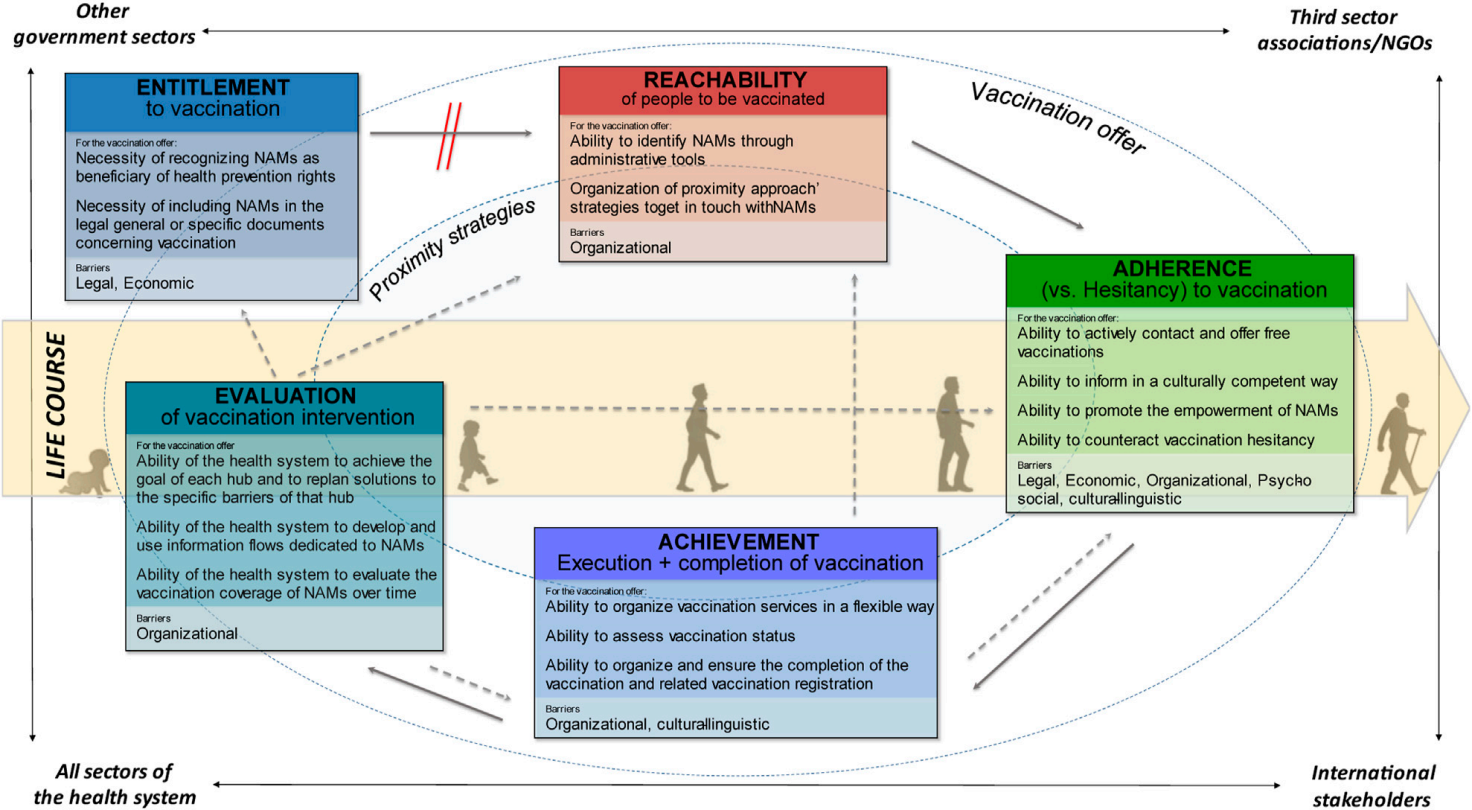
Schematization of the GCF with the specific abilities and barriers of each hub, EU/EEA, 2022.

After the PCF has been filled in and critically reviewed with the results of the non-systematic literature review and FGs and Pis, the GCF is no longer just a logical framework, but becomes a pathway that can actually strengthen health systems and make vaccination more guaranteed and equitable. Importantly, we need to move from a neutral reading to a critical re-reading, so that the GCF is no longer just a diagram. Despite the heterogeneity and breadth of the results of the analytical work, an attempt has been made in Figure 3 to graphically summarize the overall picture obtained, integrating some elements with respect to the starting outline.

#### Health System’s Strategies to Address Vaccination Barriers for NAMs by Hub

Based on literature review and experiences of health professionals, addressing vaccination barriers for migrants requires a comprehensive and multi-faceted approach. This approach should consider various factors and challenges. The following strategies are essential for overcoming system barriers and promoting successful vaccination interventions for migrants.

Entitlement to vaccination—this hub focuses on the rights of NAMs to receive vaccinations and the barriers that may arise in relation to their entitlement.

• Enhancing professional’s knowledge about health rights: Healthcare professionals must be well-informed about migrants’ rights and entitlements to healthcare services. This includes providing continuous training for healthcare personnel and practitioners on the entitlement to health and vaccinations for different profiles of migrants. Furthermore, national, regional, and local authorities should revise their immunisation policies and documents to explicitly mention migrants and NAMs as beneficiaries. This can be done by updating existing documents or creating new ones that clearly state the rights and access to immunisation services for all individuals residing within the country, regardless of their legal status or country of origin.

Reachability of people to be vaccinated—this hub addresses the challenges associated with reaching NAMs and ensuring that they have access to vaccination services. It involves proximity strategies to overcome geographical, logistical, and communication barriers.

• Improving reachability through updated data sources and multi-sector collaboaration: Accurate and up-to-date information about migrant populations is essential for effective outreach. This includes improving data sources by aligning with municipal registers and establishing links between different government departments and the National Health System (NHS). Strengthen cooperation between different levels and sectors, including general practitioners, family paediatricians, clinics for temporarily or undocumented foreigners, family planning units, hospitals, municipalities, non-governmental organizations (NGOs), and other entities that interact with migrants. Involve foreign communities in reaching out to migrants not listed for vaccination.

Adherence to vaccination—this hub deals with factors that influence the adherence of NAMs to the recommended vaccination schedule. It includes considerations like cultural beliefs, language barriers, vaccine acceptance as well as organizational vaccination literacy.

• Promoting adherence through culturally sensitive health campaigns and strategies: Plan health promotion campaigns specifically targeting migrant populations. Provide training to healthcare professionals, including language mediators, on migrant health needs, cultural awareness, vaccination hesitancy, risk communication, and community engagement. Involve migrant communities in promoting vaccinations. Strengthen links with general practitioners and family paediatricians to promote vaccinations for the entire household. Ensure all services that interact with migrants can provide accurate information on vaccination pathways.

Achievement of vaccination—this hub encompasses the actions needed to successfully administer vaccinations to NAMs, including effective delivery, monitoring, and follow-up. It involves strategies to ensure that the vaccination process is completed for each individual.

• Achieving vaccination coverage through flexible services and better documentation: Develop detailed procedures for the entire vaccination cycle and make them available to vaccine service operators. Improve access to vaccinations by extending days and time slots, facilitating reservation systems, establishing multidisciplinary teams at vaccination centers, setting up local clinics and temporary hubs, and organizing vaccination programs with support from migrant communities and third-sector organizations. Provide necessary support for migrants during the vaccination process, such as linguistic-cultural mediators and information sheets in multiple languages. Enhance communication and relational skills of healthcare professionals in the vaccination field. Facilitate the exchange of vaccine information and certificates between countries, including the availability of vaccination certificates in multiple languages.

Evaluation of the intervention—this is a cross-cutting hub which focuses on assessing the effectiveness of vaccination interventions for NAMs. It involves monitoring and evaluation activities to measure the impact of the implemented strategies and identify areas for improvement.

• Evaluating interventions through research and monitoring: Conduct surveys or focus groups to understand why certain groups do not benefit from vaccination. Monitor and evaluate the system’s ability to reach migrants, including obstacles and determinants. Study vaccine hesitancy and refusal among migrants, including obstacles and determinants. Analyze vaccination coverage for migrants and strengthen cooperation with local registries and government entities to improve data quality. Develop ways to record additional or booster doses for migrants not enrolled in the NHS.

#### Strategies Common to More Than One Hub

Certain strategies can simultaneously address the purposes of different hubs and fulfill unique purposes concurrently. The following strategic lines encompass multiple hubs:

a) *Proximity strategies*: public health strategies focus on fostering relationships between public institutions, private social organizations and communities with the goal of promoting access to services. Key features include networking, a multidisciplinary approach, the use of mobile teams, cultural mediators, and raising provider awareness through the active offer of health services, the orientation to services, the creation of pathways for taking in charge and the involvement of the population in empowerment processes. These strategies help to overcome geographical barriers and encourage better access to healthcare for migrants. Proximity strategies inform the vaccination process and are tailored to each hub, characterizing specific actions related to reachability, adherence and achievement.
b) *Training courses for providers*: the skills and competencies of providers involved in the vaccination process are crucial. Continuous training and updating, are essential for strengthening the health system and other sectors involved in vaccination. In Germany, the Robert Koch Institute (RKI) offers a range of training courses and resources for healthcare providers involved in vaccination services. These courses include topics such as vaccination schedules, vaccine storage and handling, and communication skills to effectively engage with diverse populations, including migrants. Training improve the system’s ability to reach NAMs, especially those in the hard-to-reach groups (reachability), offer and promote vaccinations, particularly also in countering vaccination hesitancy (adherence), and carry out vaccinations (achievement). Training is also a key element in developing proximity strategies should involve all stakeholders involved in the vaccination process.
c) *Migrant sensitive approach*: effective vaccination promotion, organization, and delivery require sensitivity to the unique differences of migrants, including age, gender, legal status, economic status, and more. Investing in multidisciplinary and multi-sectoral training and retraining helps strengthen the migrant-sensitive approach. In the United Kingdom, the NHS has initiated several programs to improve vaccination rates among migrants, such as offering translated materials in various languages, providing cultural competency training for healthcare providers, and using community leaders to promote vaccine uptake.
d) *Data source*: improving vaccination data collection among migrant populations is critical for developing appropriate health policies and services. Hub-specific Standardized Operating Procedures (SOPs) can enhance the quality of the vaccination process by facilitating information sharing within systems at various level. Access to disaggregated data enables better estimation of vaccination coverage, strengthens evaluation for each identified hub and improve the vaccination process in that hub. Particularly important is the registration of vaccinations and linking national and supranational databases to address the mobility of migrant populations. In the European Union, the ECDC has developed a project called “Vaccine Schedule” that gathers information on vaccination schedules and policies across EU/EEA countries. This project aims to improve data collection and sharing between countries to better understand vaccination coverage and gaps in migrant populations.

## Discussion

The purpose of this study was to develop a General Conceptual Framework (GCF) to understand how to improve the vaccination coverage for NAMs in EU/EEA. The GCF can help improve vaccination coverage for NAMs by providing a systematic approach to identifying and addressing barriers throughout the vaccination process. The methods used to identify barriers and solutions in the study were a combination of literature review and qualitative research. The literature review involved analysing relevant publications and categorizing the barriers and solutions into different subcategories. The study examined legal, economic, organizational, psycho-social, and cultural/linguistic barriers and strategies associated with entitlement, reachability, adherence, achievement, and evaluation. Additionally, qualitative research was conducted through focus groups and interviews with health and social care professionals in Germany, Poland, Spain, Italy, Greece, Malta, and Cyprus. These participants provided valuable insights into the implementation of vaccinations for NAMs and contributed to the identification of barriers and potential strategies to overcome them. The findings from both the literature review and qualitative research were used to develop the GCF and inform the recommendations for improving vaccination coverage for NAMs. The five conceptual hubs provide a framework for understanding the different stages and challenges in improving vaccination coverage adopting also a life‐course approach to immunization for children, adolescents, adults and elderly.

The framework especially helps in identifying specific barriers and possible strategies at each stage of the vaccination process. For example, it can highlight issues related to entitlement, such as ensuring that healthcare worker understand migrants’ rights to receive vaccinations and have their access to vaccination services [3, 19]. It can also help in addressing challenges related to reachability, such as improving outreach efforts to inform migrants about available vaccinations and locations [4, 81]. Furthermore, the GCF can assist in promoting adherence to vaccination by identifying strategies to overcome language and cultural barriers that may affect vaccine acceptance [17, 74]. It can also support the achievement of vaccination goals by recommending interventions like proximity interventions or organizational flexibility which bring vaccination services closer to migrant communities [47]. In terms of evaluation, the GCF emphasizes the importance of monitoring and assessing the effectiveness of vaccination interventions for NAMs [4]. This allows for continuous improvement and the identification of best practices and approach that can be shared across countries. By implementing these strategies and addressing various barriers, successful vaccination interventions for NAMs can be achieved. This will not only ensure their health and wellbeing but also contribute to the overall health of the communities they live in.

However, the study acknowledges the lack of international agreement on the definition of NAMs, which poses limitations in data collection and in understanding and addressing their specific needs. The AcToVax4NAM project has developed an operational definition of NAM based on public health considerations and guidelines, with the aim to include a wide range of people on the move, regardless of the person’s legal status or country of origin. It takes into account all ages and emphasizes the ability of the healthcare system to assess vaccination status and, if necessary, provide appropriate vaccinations. Although it was developed in a project for NAMs, we have also realized through the literature review and focus groups that the GCF can also be used for migrants who have been in the country for a longer period of time.

The study also calls for collaboration with other European projects to extend consensus on the definition of NAMs and create a greater network among countries. In fact, although the applicability of the GCF may vary across countries due to differences in healthcare systems, definitions of NAMs, and other contextual factors, the framework provides a starting point for addressing vaccination coverage challenges. By comparing the findings of the GCF with relevant literature and adapting it to specific country contexts, policymakers and healthcare providers can tailor their strategies to improve vaccination coverage for NAMs effectively. Based on our extensive literature review, we have found no existing conceptual framework that precisely outlines the vaccination process for NAMs, as illustrated in this study.

This study also suggest to create and adapt SOPs and country specific action-oriented flow-chart by drawing from the 5-hub of the GCF. SOPs should cover all aspects of vaccination and be implemented at the national level by all relevant health services. This will allow all stakeholders involved to know what to do, how to do it and with whom it is important to collaborate, as there is not one-size-fits-all model for national health systems or migrants’ rights. Finally, advocating for migrants’ rights to healthcare and promoting inclusive policies can help create a supportive environment for vaccination efforts. Engaging policymakers, civil society organizations, and other stakeholders can lead to the development of policies that prioritize migrants’ health and wellbeing.

In conclusion, vaccinations play a crucial role for NAMs, as they may be a vulnerable group in need of protection from infectious diseases. During their journey and settlement in a new community, migrants can be exposed to poor hygiene conditions and limited access to medical care, increasing their vulnerability to infectious diseases. Vaccinations represent an effective means to prevent the spread of infectious diseases within migrant populations, protecting not only the migrants themselves but also the host communities. Ensuring that migrants have equal access to immunization services reduces the risk of outbreaks and the spread of diseases within communities. Furthermore, it promotes the wellbeing and integration of migrants, leading to healthier and more inclusive societies. Overall, the GCF developed in this study will serve as a blueprint for each country to develop their own action-oriented flow charts/SOPs based on the specific barriers and proposed strategies identified. The framework will help improve vaccination coverage for NAMs, taking into consideration different NAMs categories, the heterogeneity of settings, age groups, and special groups such as pregnant women. Countries should define specific actions, responsible parties, and timelines in their unique contexts, while reflecting on the necessary resources.
