# Supplementary material for: Access to Vaccination for Newly Arrived Migrants: Developing a General Conceptual Framework for Understanding How to Improve Vaccination Coverage in European Countries
Source: Int J Public Health. 2023 Aug 7;68:1605580. doi: 10.3389/ijph.2023.1605580 (PMC10440383; doi:10.3389/ijph.2023.1605580)
Supplement: Supplementary file 1 [file DataSheet1.docx]

## Supplementary Material 1

Question Groups

GCF hubs (description and question groups)

| **GCF Hub/concept** | **Description** | **Question Groups** |
| --- | --- | --- |
| **ENTITLEMENT**  to vaccination  **WHAT** rights for vaccination to **WHOM?** | This hub concerns the regulatory planning of the vaccination offer.  **If there is NO entitlement the process CANNOT start.** | **Legal barriers/solutions**   - Is there a national vaccination plan?   - If so, does this plan consider NAMs?   - If so, which categories of NAMs are covered?   - If not, are there other documents considering NAM vaccination? - Are NAMS entitled to vaccinations?   - What are the differences between the different categories of NAMs regarding vaccination entitlement?   - Are there any differences with respect to the local population when it comes to entitlement to vaccination?   **Economic barriers / solutions**   - Does the Health System require the full payment of vaccinations by NAMs?   - Are there any differences between the different categories of NAMS regarding payment of vaccination? - Does the Health System require a co-payment fee for NAMs vaccinations?   - Are there any differences between the different categories of NAMs regarding vaccination co-payment fees? |
| **REACHABILITY**  of people to be vaccinated  **HOW** (the health service gets in contact with) **to** **WHOM** (NAMs)? | This concept regards all strategies, including the ‘proximity approach’, and abilities of the health service to get in contact with NAMs | **Organizational barriers / solutions**   - Where are NAMs staying (centres, camps, community)? - Are there any differences between the different categories of NAMs? - Are there any lists of NAMs who arrived in the country in the previous 12 months? Where is it possible to get this information? - Are there any differences between the different categories of NAMs? - Through which channels are NAMs contacted? Are NAMs contacted at an individual / collective level (e.g. reception centres)? - Are there any differences between the different categories of NAMs? - Is there a possibility for NAMs or NGOs working with NAMs to actively request vaccinations? - Are there any differences between the different categories of NAMs? - Are health, social and police workers adequately trained regarding NAMs vaccination rights? |
| **ADHERENCE**  (vs. Hesitancy)  to vaccination  **HOW** (vaccines are offered) **for WHAT** (NAMs needs)? | This concept includes strategies to ensure that NAMs respond positively to the vaccination offer. It is also necessary to devise strategies and abilities in the professional FOR health to counteract vaccination hesitancy, fear, and other psycho-social issues among NAMs | **Legal barriers/solutions**   - Is there a reporting obligation / risk to non‐sanitary bodies (and in particular to the Police) for undocumented NAMs?   **Economic barriers / solutions**   - Is the vaccination offer truly free of any charge, if the NAM is entitled to?   **Organizational barriers/solutions**   - Is the vaccination offer ‘active’ (in the sense of actively proposed by the Health System and not only consequent to a specific request of the NAM)? - Are the services easily accessible? - Is the vaccination offer organized with proximity services?   **Psycho-social barriers/solutions**   - Is the fear of reporting to non-sanitary bodies by the vaccination services countered? - Are the vaccinations voluntary, confidential, non‐stigmatizing?   **Cultural‐linguistic barriers/solutions**   - Is adequate and accessible information (by method and language) provided by the vaccination service? - Is adequate and culturally competent information provided about the importance and safety of vaccination to improve adherence? - Which communication channels are used (health professionals, community leaders, law enforcement agencies, etc.)? - Are interventions being implemented to combat fake news on vaccines? - Are health, social and police workers adequately trained to address the cultural barriers so as to ensure an adherence to vaccination? |
| **ACHIEVEMENT** Execution + completion  of vaccination  **WHAT** (vaccines) **and WHEN for WHOM** and **HOW for WHAT?** | This concept concerns the execution and the completion of vaccination and should focus on organization and flexibility of health services. | **Organizational barriers/solutions**   - Is there an assessment of the previous vaccination status of individual NAM? How is this assessment done? - Are vaccination services flexible in terms of organization / time? - Do NAMs have easy physical access to vaccination services? - Is a vaccination certificate issued? - How are the vaccinations carried out recorded? - Are there any differences between the different categories of NAMs in the organization of the vaccine process?   **Cultural-linguistic barriers/solutions**   - Is informed consent understandable (simple and translated into a language known by the NAMs)? - Are health professionals properly trained to address linguistic and cultural barriers? Is there any specific training available for professionals? - Are cultural mediators available for the needs of the service? |
| **EVALUATION**  of vaccination intervention  **HOW many** and **WHO** (vaccinated) **among ALL (**NAMs**)**. | This concept stresses the importance of the vaccination evaluation through every step of the vaccination process. It is linked to every other hub as the dashed arrows shown in figure 4. The vaccination Evaluation needs to involve the strategies and actions of every other previous hub. | **Organisational barriers/solutions**   - Does the Health System have an information flow dedicated to vaccinations at national level? - Does the flow allow the extraction of data for NAMs? - Is it possible to calculate the vaccination coverage for NAMs? - Is vaccination data available as a numerator of vaccination coverage?   - Is there a local/national database?   - How long has the data been collected? - Is the data of NAMs that entered the country in the previous 12 months available as a denominator of vaccination coverage?   - Is there a local/national database?   - How long has the data been collected? - Are there any differences between the different categories of NAMs |

## Supplementary Material 2

Desktop Literature review search strategy including inclusion and exclusion criteria

**Protocols for desktop non-systematic review to identify system barriers (legal, linguistic, cultural, logistic) to immunization of NAMs and solutions already implemented to overcome**

**WP4 – Immunization guidance, reception & vaccine offer systems for NAMs and related barriers: a conceptual framework**

The work of the WP4 will guide the implementation of the subsequent WPs. It is a crucial step for the project as during this phase an updated description of the existing situation in each of the consortium countries concerning the reception systems and vaccination systems for NAMs will be provided. The consortium will conduct desktop review and original research (qualitative participatory research) to identify country specific system barriers and relevant solutions. The results will be critically analysed and organized in a General Conceptual Framework, intended to be useful also for other EU countries not participating to the project. The WP4 has the following two Specific Objectives.

Specific Objective 1: To describe existing immunization guidance and the reception and vaccination offer systems for NAMs in consortium countries

Specific Objective 2. To characterize the specific system barriers that hinder the immunization of NAMs and propose possible solutions

The work of this WP is divided into 5 tasks, Task 4.1 relates to the Desktop review object of this protocol.

**Task 4.1 Desktop review on guidance for and barriers to immunization of NAMs**

Desktop non-systematic review of European and national level guidance and recommendations (WHO, ECDC, peer reviewed publications and grey literature) on immunization offer and practice for NAMs. The review will also search for barriers to the immunization of NAMs. The WP leader, with the support of a panel of subject matter experts from the consortium, will develop a protocol that will include the definition of system barriers and in particular the definition of legal, linguistic, cultural and logistic barriers, caused by the social and political contexts that NAMs face. Based on these definitions, all partners will collaborate to search at country level for existing information on these aspects in their respective countries in the last 10 years. The task will provide information and guidance to be used in the subsequent WP tasks. The WP leader (ISS) will carry on the review in collaboration with partners.

In order to fulfil these tasks the following methods will be used:

**Desktop literature review of existing research**. Classical desktop literature review will be implemented in order to describe the **state of the art of guidance and practice recommendations** on immunization of NAMs at European level and in each consortium country.

**Desktop literature review** will be implemented, also at country level, to identify existing research concerning **system barriers** at legal, linguistic, cultural and logistic level **and solutions** already implemented to overcome them.

**Non-systematic review protocol of**

**system barriers (legal, linguistic, cultural, logistic) to immunization of NAMs**

**and solutions already implemented to overcome them**

In addition to the barriers (legal, linguistic, cultural, logistical, etc.), the review will consider solutions to overcome these barriers and implementation challenges.

A specific section of the search will be dedicated to the analysis of specific policies and practices in place for vaccination against COVID-19 disease. As such, campaigns present different and specific characteristics and challenges it is of relevance to be able to document in the results what has emerged in terms of specific approaches proposed and/or implemented to ensure equity in terms of access to COVID-19 vaccines. In addition, it is thought that given the specific push to consider underserved populations in times of the pandemic, a number of very critical lessons could be learnt that can be transferred to other routine vaccinations as well, applying the same tools/methods that may have worked for vaccination against COVID-19. As the latter is high on the political agenda, suggestions could be made from the analysis as to how policy approaches could change and learn/be adapted from the recent experiences.

**a) system barriers to immunization of NAMs and solutions implemented**

**Research strategy for Medline:** (immunization OR vaccination OR vaccination plan OR vaccine) AND ((migrant OR newly arrived migrants OR undocumented migrants OR irregular migrants OR illegal migrants OR asylum seekers OR foreigners) OR (regular migrants OR documented migrants OR refugees)) AND (barriers OR access OR obstacles OR difficulties OR accessibility OR utilization OR delivery OR uptake OR supply OR hard-to-reach OR equity OR acceptance OR hesitancy OR facilitator OR solution OR effectiveness OR best practice)

**Keywords for other websites**: immunization, vaccination, vaccine, migrants, asylum seekers, refugees, foreigners, barriers, access, obstacles, difficulties, accessibility, utilization, delivery, uptake, supply, hard-to-reach, equity, hesitancy, solution, effectiveness, best practice

**b) Focus on COVID-19 vaccination**

**Research strategy for Medline:** (immunization OR vaccination OR vaccination plan OR vaccine) AND ((migrant OR newly arrived migrants OR undocumented migrants OR irregular migrants OR illegal migrants OR asylum seekers OR foreigners) OR (regular migrants OR documented migrants OR refugees)) AND (barriers OR access OR obstacles OR difficulties OR accessibility OR utilization OR delivery OR uptake OR supply OR hard-to-reach OR equity OR acceptance OR hesitancy OR facilitator OR solution) AND (COVID-19 OR Sars-Cov-2)

**Keywords for other websites:** immunization, vaccination, vaccine, migrants, asylum seekers, refugees, foreigners, barriers, access, obstacles, difficulties, accessibility, utilization, delivery, uptake, supply, hard-to-reach, equity, hesitancy, solution, effectiveness, best practice, COVID-19, Sars-Cov-2

| **Website to explore** | |
| --- | --- |
| - MedLine-PubMed, for a search in the scientific literature | - EC - European website on integration |
| - WHO | - EC – European Observatory on Health Systems and Policies |
| - WHO Europe | - EUPHA |
| - WHO PHAME Public Health Aspects of Migration in Europe | - PICUM (Platform for International Cooperation on Undocumented Migrants) |
| - UNHCR | - Reliefweb |
| - UNICEF | - COVAX |
| - United Nations High Commissioner for Human Rights (OHCHR) | - GAVI |
| - CMW (UN Committee on Migrant Workers) | - MSF Medicins sans Frontieres |
| - UN Special Rapporteur on the Human Rights of Migrants | - Project PROMOVAX <http://www.promovax.eu/> |
| - IOM | - Project CARE–Common Approach for REfugees and other migrants’ health” <http://careformigrants.eu/> |
| - ECDC | - Mig-HealthCare Project <https://www.mighealthcare.eu/> |
| - Council of Europe | - Project MyHealth <http://www.healthonthemove.net/it/> |
| - European Commission |  |

**Inclusion Criteria**

The following criteria, not included in the search strings, should be applied during the selection of relevant documents to be selected:

- **Time limits**: since 2014 when, given the larger influx of NAMs in EU, relevant analyses and documents in the field were available in the European context, taking into consideration only the most updated documents in presence of several documents of the same kind.
- Documents included must refer to **newly/recently arrived migrants** based on the definition used for the project*.*
- Documents must refer on **EU Countries.** However, as regards the barriers and possible solutions, it will also be useful to examine documents referring to non-EU developed countries, which could present situations applicable also in the EU context.

**Division of Task between WP4 leader and consortium partners**

The WP4 leader will perform the literature and document review in English, while the consortium partners will conduct the review in local language as explained in the next paragraph.

**Guidelines for consortium partners**

In order to find scientific articles or documents not accessible in English, each referent of the **consortium Countries will be asked** to integrate the search with materials in local languages or contained in websites not taken under consideration.

The **kind of documents** in local language to be searched for are:

- Scientific literature
- Guidance
- Guideline
- Bulletin
- Report
- Legislation
- Policy document
- Standard operating procedure

The keywords, to be translated in local language, to guide the search are:

**For system barriers to immunization of NAMs and solutions implemented**

immunization, vaccination, vaccine, migrants, asylum seekers, refugees, foreigners, barriers, access, obstacles, difficulties, accessibility, utilization, delivery, uptake, supply, hard-to-reach, equity, hesitancy, solution, effectiveness, best practice

**for the focus on COVID-19 vaccination**

immunization, vaccination, vaccine, migrants, asylum seekers, refugees, foreigners, barriers, access, obstacles, difficulties, accessibility, utilization, delivery, uptake, supply, hard-to-reach, equity, hesitancy, solution, effectiveness, best practice, COVID-19, Sars-Cov-2

**Example of websites to be consulted for Italy**

- Scientific articles in Italian non indexed in Medline
- Italian National Institute of Health (ISS)
- Epidemology for Public Health (EpiCentro-ISS)
- National Institute for Health, Migration and Poverty (NIHMP)
- Italian Ministry of Health
- Italian Society of Migration Medicine and Congress Proceedings
- Regional Health Authorities
- Salute Internazionale
- Osservasalute
- Rapporto IDOS
- Civil Society organizations: ASGI, Emergency, Centro Astalli, Intersos, Medici contro la Tortura, Médecins du Monde, MEDU, Medici Senza Frontiere, Caritas italiana, Sanità di Frontiera.

In order to transfer to WP4 leader (ISS) relevant information from the consortium Countries, **an information extraction grid in Excel is provided.**

All the information to be filled in should be translated in English.

The Excel file has the following columns (information) to be filled in:

| **Column name** | **Description** |
| --- | --- |
| Document progressive number | Progressive identification number of the specific document |
| Country | Name of the consortium Country |
| Reviewer | Name of the operator who is conducting the search |
| Topic | - RECOMMENDATIONS - BARRIERS/SOLUTIONS - RECOMMENDATIONS/BARRIERS/SOLUTIONS |
| Title | Title of the document |
| Authors | Authors of the document |
| Year | Publication year |
| Publisher | Institution that published the document |
| Source/website | Consulted website |
| DocumentType | - Scientific literature - Guidance - Guideline - Bulletin - Report - Legislation - Policy document - Standard operating procedure - Other document type |
| Abstract/Summary | If available, copy/paste of the abstract/summary in English |
| Link to full text | Link to full text of the document |
| Population | Specify the subpopulation considered in the document (among NAMs as defined within the project) |
| Setting | Specific setting taken under consideration in the recommendation or in the description of a barrier/solution |
| Vaccination | Type of vaccination target of the document (i.e. all compulsory vaccination, or a specific vaccine: COVID-19, MMR, HPV, HBV …) |
| Recommendation | The recommendation to vaccination included in the document (one recommendation for each record of the Excel file) (max 100 words) |
| Comments | Any useful comment from the reviewer |

**NOTE THAT** since each document can contain more than one barrier or solution, you must use one line of the Excel file for each recommendation or barrier or solution contained in the same document.

Only in the case that the elements (barriers / solutions) of a document are linked, they must be written on the same line of the excel file.

## Supplementary Material 3

**Table 1** Studies which reported at least a barrier (x) and/or a solution (o) by GCF hub

| ***Ref*** | ***Studies*** | ***Entitlement*** | | ***Reachability*** | | ***Adherence*** | | ***Achievement*** | | ***Evaluation*** | |
| --- | --- | --- | --- | --- | --- | --- | --- | --- | --- | --- | --- |
| ^11^ | *Abdi, 2019* |  |  |  |  | x | o |  |  |  |  |
| ^12^ | *ALISA, 2021* |  |  |  |  |  |  |  | o |  |  |
| ^13^ | *Allen, 2019* |  |  |  |  | x | o |  |  |  |  |
| ^14^ | *Armocida, 2021* | x |  |  |  |  | o | x | o |  |  |
| ^15^ | *Bandini, 2021* |  |  | x | o |  | o | x | o |  |  |
| ^16^ | *Bartovic, 2021* |  |  |  |  | x | o | x |  | x | o |
| ^17^ | *Bradby, 2015* |  |  |  |  |  |  | x |  |  |  |
| ^18^ | *Crawshaw, 2021* | x | o |  |  | x | o |  |  |  | o |
| ^19^ | *Dalla Zuanna, 2018* |  |  |  |  |  |  | x |  | x | o |
| ^20^ | *De Vito, 2017* | x |  |  | o | x | o | x | o |  | o |
| ^21^ | *Deal, 2021* |  |  |  |  | x | o |  |  |  |  |
| ^22^ | *Declich, 2021* |  |  |  |  | x | o | x | o |  |  |
| ^23^ | *Del Manso, 2017* |  |  |  |  |  |  | x |  |  |  |
| ^24^ | *Driedger, 2018* |  |  |  |  | x | o |  |  |  |  |
| ^25^ | *Epicentro, 2021a* |  |  |  |  |  | o |  |  |  |  |
| ^26^ | *Epicentro, 2021b* |  |  |  |  |  | o |  |  |  |  |
| ^27^ | *ECDC – Scientific advice, 2015* |  |  |  | o |  |  | x | o |  |  |
| ^5^ | *ECDC Scientific advice, 2018* | x |  | x |  | x | o | x | o |  |  |
| ^28^ | *ECDC, 2021a* |  |  | x |  | x | o |  | o |  |  |
| ^29^ | *ECDC, 2021b* | x | o |  |  | x | o |  |  |  |  |
| ^30^ | *Fabiani, 2017* |  |  |  |  | x | o |  |  |  |  |
| ^31^ | *Garcia Galan, 2007* |  |  |  |  |  |  | x | o |  |  |
| ^32^ | *Geraci, 2021* |  |  |  |  |  |  | x | o |  |  |
| ^33^ | *Ghebrendrias, 2021* |  |  |  |  | x | o |  |  |  |  |
| ^34^ | *Giambi, 2017* |  |  | x | o | x |  | x | o |  |  |
| ^2^ | *Giambi, 2019* | x |  | x | o |  |  | x | o |  |  |
| ^35^ | *Godoy-Ramirez, 2019* |  |  |  |  | x | o | x |  |  |  |
| ^36^ | *Hargreaves, 2018* |  |  |  |  | x |  |  |  |  |  |
| ^37^ | *Huerta González, 2016* |  |  | x | o |  | o |  | o |  |  |
| ^38^ | *Hui, 2018* |  |  |  | o |  |  | x |  |  |  |
| ^39^ | *IOM, 2021* | x |  |  |  | x |  | x |  |  |  |
| ^40^ | *Italian MoH, 2017* |  |  | x | o |  | o | x | o |  |  |
| ^41^ | *Karnaki, 2018a* |  |  |  |  |  |  | x |  |  |  |
| ^42^ | *Karnaki, 2018b* |  |  |  |  |  |  | x | o |  |  |
| ^43^ | *Knights, 2021* |  |  | x | o | x | o |  |  |  |  |
| ^44^ | *Krishnaswamy, 2018* |  |  | x | o | x | o | x | o |  |  |
| ^45^ | *Mahimbo, 2017* |  |  |  |  |  |  | x | o |  |  |
| ^46^ | *McComb, 2018* |  |  |  |  | x |  |  |  |  |  |
| ^47^ | *Mellou, 2019* |  |  |  |  |  |  | x | o |  |  |
| ^48^ | *Mipatrini, 2017* |  |  |  |  | x | o | x | o |  | o |
| ^49^ | *Noori, 2021* | x | o |  |  | x |  | x | o | x | o |
| ^50^ | *Ortiz, 2020* |  |  |  |  | x | o |  | o |  |  |
| ^51^ | *Pavli, 2017* | x | o | x | o | x | o | x | o |  |  |
| ^52^ | *Perry, 2020* |  |  |  |  | x |  | x |  |  |  |
| ^53^ | *PICUM, 2021* | x |  |  |  |  |  | x |  |  |  |
| ^54^ | *Prymula 2018* |  |  |  |  | x |  | x | o |  |  |
| ^55^ | *Razum, 2020* | x |  |  |  | x |  |  |  |  |  |
| ^56^ | *Rechel, 2019* |  |  | x |  | x | o |  | o |  |  |
| ^57^ | *Regione Toscana 2019* |  |  |  |  |  |  | x | o |  |  |
| ^58^ | *Riza, 2018* |  |  |  |  |  | o | x |  |  |  |
| ^59^ | *Robert Koch-Institut Bulletin, 2020* |  |  |  |  |  |  | x |  |  |  |
| ^60^ | *Rubens-Auguston, 2019* |  |  |  |  | x | o | x |  |  |  |
| ^61^ | *Shetty, 2019* |  |  |  |  | x | o |  |  |  |  |
| ^62^ | *Socha, 2020* |  |  |  |  | x | o | x |  | x | o |
| ^63^ | *Spallek, 2019* | x | o |  |  |  |  |  |  |  |  |
| ^64^ | *Sypsa, 2016* |  |  | x |  | x |  | x |  |  |  |
| ^65^ | *Tavolo Immigrazione Salute TIS e Tavolo Asilo e Immigrati TAI, 2021* |  |  | x | o |  |  |  |  |  |  |
| ^66^ | *Tavolo Asilo e Immigrazione, 2021* |  |  |  |  | x | o |  |  |  |  |
| ^67^ | *Teerawattananon, 2021* |  |  |  |  | x | o |  |  |  |  |
| ^68^ | *Theodorou, 2012* | x | o |  |  |  |  |  |  |  |  |
| ^69^ | *Thomas, 2021a* |  |  |  |  | x | o | x | o |  |  |
| ^69^ | *Thomas, 2021b* |  |  |  |  |  | o |  |  |  |  |
| ^70^ | *Tosti, 2021* |  |  |  | o |  |  |  |  |  |  |
| ^71^ | *Vita, 2019* |  |  |  |  | x |  |  | o |  |  |
| ^72^ | *WHO-UNICEF, 2021* |  |  |  |  |  |  | x |  |  |  |
| ^73^ | *WHO-UNHCR-UNICEF, 2015* | x | o |  |  | x | o | x | o |  |  |
| ^74^ | *Williams, 2016* |  |  | x |  |  | o |  |  |  |  |
| ^75^ | *Wilson, 2018* |  |  |  | o | x | o |  | o |  |  |
| ^76^ | *WHO Europe, 2014* | x | o |  | o | x | o |  | o |  | o |
| ^77^ | *WHO Europe, 2017* | x |  |  |  |  | o |  | o |  |  |
| ^78^ | *WHO Europe, 2018a* | x |  |  |  |  |  | x |  |  |  |
| ^79^ | *WHO Europe, 2018b* |  |  |  | o | x | o |  |  |  |  |
| ^80^ | *WHO Europe, 2018c* |  |  |  |  |  | o |  | o |  |  |
| ^4^ | *WHO Europe, ‎2019a* | x |  | x | o | x | o | x | o | x |  |
| ^81^ | *WHO Europe, 2019b* |  |  | x | o | x | o | x | o |  |  |
| ^82^ | *WHO Europe, ‎2019c‎* |  |  |  |  | x |  | x | o |  |  |
| ^83^ | *WHO Europe, 2020* |  |  |  |  |  |  |  |  |  | o |
| ^84^ | *Zard, 2021* |  | o | x | o | x | o | x | o |  |  |

## Supplementary Material 4

Guidelines for performing FGs discussion and PIs

**Focus Group/Interview Guide**

**SUMMARY**

This document defines the framework for implementing the qualitative research planned within WP4 of the “AcToVAx4NAM” project and more specifically for organizing Focus Groups (FGs) and Personal Interviews (PIs).

The qualitative research is an added value to the project, which will ensure that final solutions and recommendations will be based not only on the description of the systems and the published materials (as per Task 4.1, 4.2 and 4.3) but also on experiences of the target group (here called “professionals FOR health”). The aim is to achieve the characterization of system barriers (legal, linguistic, cultural, and logistic, etc.) and identification of possible and sustainable solutions emerging through a participatory approach.

This guide sets the objectives, expected outcomes, and indicative guidelines for the local organizers and facilitators of FGs and PIs in all participating countries. More specifically, it intends to provide uniform guidance for the facilitators/moderators, in order to be able to conduct the FGs and PIs analyze the data and report the findings.

**1. Focus groups and personal interviews**

The aim of the FGs and PIs is to gain insight into the experiences of the participants – meaning here professionals FOR health who work with Newly Arrived Migrants (NAMs) or migrants in general in order to:

1. identify and understand system barriers towards NAMs’ immunization (legal, linguistic, cultural and logistic),
2. explore possible and sustainable solutions at country level.

Emphasis will also be placed on the consequences of COVID-19 for the vaccination of NAMs and also on the opportunity COVID-19 has provided in the field of NAMs’ immunization, if any.

During the FGs and PIs we want to deepen into (a) the experiences of professionals involved with NAMs’ immunizations, and (b) the necessary cooperation between involved actors, concerning identified difficulties, and the tested or/ and proposed solutions.

According to the AcToVax4NAM project operational definition, “NAMs” include:

- Documented migrants, asylum seekers, refugees, and others forced to flee conflict, natural disasters or economic peril, as well as undocumented migrants
- A person with a different citizenship from the hosting country either from EU/EFTA Member States or Third countries
- Entered the EU consortium country in the last 12 months, (excluding tourists and short visa and permit of < 3 months) plus 12 months in case of relocation or transfer from EU countries

**2. Timeline**

All partners must submit their FGs and PIs reports to **Prolepsis Institute on Friday, January 14, 2022.**

**3. Number of focus groups and personal interviews**

**Each partner country** will conduct:

- **2 focus groups, and**
- **3 personal interviews.**

**4. Duration of focus groups and personal interviews**

- The duration of each Focus Group will be approximately 45-90 minutes.
- The duration of each personal interview will be approximately 30-40 minutes.

The above time durations are indicative and depend on the country specific circumstances.

**5. Participants**

*Number of participants*

The number of participants in each FG will be **8-10**. Therefore, the total number of FG participants per country in the qualitative study will be **16-20**. However, the recruitment of participants can be a very demanding process, hence the optimal number may be difficult to reach and therefore, each FG should aim to reach at least 6-8 participants. Yet, we are trying for the optimum participation that is, 8-10.

The total number of personal interview’ participants per country in the qualitative study is **3**.

**The optimal number of participants in the AcToVAx4NAM qualitative study is 19-23.**

*Recruitment and eligible participants*

Participants can be recruited in various ways, based on the country-specific cultural and institutional context. Due to time constraints, partners could use gatekeepers and their organization’s networks. Gatekeepers are individuals or organizations who have a prominent role in each target population.

FG participants include the following groups:

- - Health and social care professionals who work in the field of delivery of immunizations with special attention to migrants if possible. Their occupation could be physicians, nurses, social workers, psychologists, cultural mediators etc.

Therefore, participants need to work in the day-to-day delivery of immunization (with special attention to migrants and NAMs if possible) and could be recruited from different locations, i.e. health centres, vaccination local/ national units, NGOs, entry camps, first reception, detention, apartments, associations, shelter centers, where both children/ adolescents and adults are cared for.

**This group of professionals constitute the participants of the 1^st^ Focus Group**

- **Professionals, who work in managing/organizing immunization services with special attention to migrants if possible.** Their occupation could be managers, administrative staff, physicians, nurses, social workers working in managing/organizing immunization services.

Therefore, participants need to work in the management, organization and administration of the immunization services to NAMs and could be recruited from different locations, i.e. health centres, vaccination local/national units, NGOs, entry camps, first reception, detention, associations, where both children/adolescents and adults are cared for.

**This group of professionals constitute the participants of the 2^nd^ Focus Group**

- - **Experts related to immunization planning with special attention to migrants if possible.** They could be policy makers, public health experts, actors involved in the development of the National Immunization Plan, migrant community leaders, etc.

Therefore, participants have a higher professional status and could be recruited from different locations, i.e. Ministries, Institutes of Health, Universities, regional health services, hospitals, International NGOs, Migrants’ organizations etc.

**This group of professionals will be the participants of the 3 Personal Interviews**

**Important considerations:**

- **Partner-countries need to choose, if they will consider the discussion on NAMs immunization at national or local level or both taking into consideration each country’s specific situation.**
- **We are interested in NAMs’ immunization throughout the life course. Therefore, it is important to recruit participants, who work both with children/adolescents and adult/elder NAMs.**
- **Another important factor is the legal status of NAMs. Therefore, it is important to recruit participants who work with the different categories of NAMs as outlined in the project NAM definition.**
  - Documented migrants, asylum seekers, refugees, and others forced to flee conflict, natural disasters or economic peril, as well as undocumented migrants
  - A person with a different citizenship from the hosting country either from EU/EFTA Member States or Third countries
  - Entered the EU consortium country in the last 12 months, (excluding tourists and short visa and permit of < 3 months) ® 12 months in case of relocation or transfer from EU countries

**6. Delivery of focus groups and personal interviews**

*On-line focus groups and personal interviews*

Due to the coronavirus pandemic and in accordance to each partner country’s safety guidelines, FGs and PIs might need to change to online focus groups and personal interviews. This modification does not alter the “classic” focus group/ personal interview methodology, which is described more thoroughly in the next chapters. Nevertheless, in the following section we have included empirical data that might be useful in case partners have to conduct online focus groups/ personal interviews:

- **Selection of an online platform:** Each partner organization is free to choose whichever platform they wish to use. Prior experience with online FGs has shown that Zoom is a reliable platform.
- **Demographics questionnaire and consent form:** both can be emailed to participants a few days before the online focus groups. For example, for FGs conducted in Greece, questionnaires translated into Greek and transferred into a Google Form platform.
- **Recorder:** Online platforms usually have the option of recording. Participants need to provide their consent before the use of the recording (consent form).
- **The role of the note taker:** The note taker/second moderator could also engage with the technical issues, since during the online focus groups technical limitations and problems might occur. The note taker/second moderator could effectively solve these issues. This way the conversation flow remains unaffected.

*Consent of participants*

Participants need to provide their consent for participation in the focus groups and personal interviews. A consent form in English is provided in **Annex 1**. The consent form needs to be translated in each partner country language. All participants need to provide a signed consent form before participating in the focus groups/ personal interview. The facilitators need to collect the signed consent forms and keep them in confidential records. For on-line focus groups/ personal interviews, the participants need to fill in the form and send it electronically.

**Important information about the consent forms (Annex 1):** the informed consent forms will be collected under the responsibility of each partner organization. Each partner organization should store them during the whole project ensuring all privacy related issues.

*Descriptive characteristics of participants*

It is important to collect some descriptive characteristics of the FG/PI participants. A descriptive questionnaire in English is provided in **Annex 2**. The questionnaire does not contain any personal information/ identifiers, such as names, e-mails etc. The questionnaire needs to be translated in each partner country language. All participants need to fill in the descriptive questionnaires before participating in the focus groups/ personal interview. The facilitators need to collect the questionnaires.

**For on-line focus groups/personal interviews**, partners could transfer the questionnaires into “google forms”. Participants will be able to fill in the questionnaires electronically.

**Important considerations about the descriptive questionnaire (Annex 2):**

1. **After the completion of the fieldwork, partners should send Prolepsis an excel file without personal identification for privacy reasons, including the data of the descriptive questionnaires.**
2. The descriptive questionnaires are provided for completion at the beginning of each focus group/ personal interview. It is crucial to collect accurate descriptive information about participants’ demographics. Demographics are important for the data analysis as well as research/publication purposes.

*Topics of the focus group discussion/personal interviews*

The main goal of the FG and PI discussions is to deepen into (a) the experiences of professionals and (b) the cooperation between the involved actors in relation with the difficulties and the tested or/ and proposed solutions related to NAMs immunization.

The topics will **focus on** **5 specific concepts/hubs** related to NAMs’ vaccination that have been presented in the “General Conceptual Framework on how to improve the vaccination coverage of NAMs” (WP2, Task 4.5, milestone 4.2):

1. ENTITLEMENT to vaccination
2. REACHABILITY of people to be vaccinated
3. ADHERENCE (vs. Hesitancy) to vaccination
4. ACHIEVEMENT of vaccination (execution and completion)
5. EVALUATION of vaccination intervention

By the end of the two (2) FGs and the three (3) PIs, the aim is to cover all concepts/hubs of the conceptual framework. It is expected that participants in each FG and in in the PIs may be more familiar with some of the concepts/hubs of the framework and not others, due to their profession or other experiences. The FGs and the PIs should focus on what the participants have to share. For example, people working in the field possibly have more insight on the concepts of reachability, adherence and achievement compared to participants in the PIs (experts/professionals of higher status) who possibly have more to say about the concepts of entitlement and evaluation. This means that during discussions participants will be given the space to emphasise more on specific concepts, based on their experiences and profession. This is desirable and should be encouraged by the moderators. Moderators during the FGs and PIs should promote the free flow of the discussion and should not press the participants with probing on each concept of the framework.

The discussion should consider the ActoVax4NAM operational definition -differences in age and legal status of NAMs. Moreover, emphasis will be given on the consequences of COVID-19 for the vaccination of NAMs and on the opportunity, COVID-19 has provided in the field of NAMs’ immunization, if any.

**One** **(1) discussion guide** has been designed (**Annex 3**) for FGs and PIs. This guide will be used by moderators to guide the discussions. The questions included In the discussion guides are **indicative.** Moderators are free to direct the discussions based on the group dynamics and the experiences of the participants asking the questions mostly appropriate.

**7. Data analysis**

All focus groups and personal interviews need to be recorded and transcribed as analysis will be conducted based on these transcriptions. Transcriptions should be in the language of each partner country. There is no need for transcriptions’ translation into English.

In order to ensure credibility and reliability of the research, the written format of the transcriptions should be an exact replica of the audio file as recorded. The recording of pauses, silences and repeated words is desirable (could also be noted by the note keeper) but depends on how each team in conducting their analysis. There is no need to send records and transcriptions to Prolepsis.

**Annex 4** includes the results template -National Report- which needs to be filled in and sent to Prolepsis. The results template describes how reporting of focus groups and personal interviews should be conducted. Quotations justifying each finding need to be included.

**8. Deliverables**

Each partner conducting the focus groups and personal interviews needs to deliver to Prolepsis Institute a national report detailing the methodology of the focus groups/ personal interviews and the results based on the results template described above (**Annex 4**). Each partner country should also send to Prolepsis Institute the three excel files including the data from the descriptive questionnaires - one for each focus group and one for personal interviews.

**The deadline is the 14^th^ of January, 2022.**

**Partners will send the country specific focus groups reports to the Prolepsis team, who will compile results and produce an overall analysis of findings and conclusions. The overall analysis will constitute the main part of the final report, which will also include each partner country’s content analysis. Therefore, please, be precise and specific in the data analysis reports.**

**9. Useful information for focus group and interview moderators**

Each focus group will have a **moderator (facilitator)**. The moderator will need to ensure maximum participation, ensure the discussion is targeted to the objectives set which should also be oriented to produce results. The role of the facilitator is crucial in conducting the focus groups effectively, especially in terms of providing clear explanations of the aim of the group discussion, helping people feel at ease, and facilitating interaction between group members. To this end, it is important that the facilitators have experience in moderating as well as good interpersonal skills in order to promote participants’ trust and increase the likelihood of an open and interactive dialogue. Α **note taker (rapporteur)** can also participate in each focus group. The main role of the rapporteur will be to keep track and take notes of the main issues discussed.

Crucial issues:

CONFIDENTIALITY

It is crucial that no one except the organizers and the participants is aware of the participants’ names. Furthermore, people other than Consortium members should **not** have access to individual participants’ answers, neither accidentally nor intentionally. Do not refer to the names of respondents in the interview notes. Use also a unique code assigned to the respondent to protect confidentiality.

BUILDING RAPPORT

- **Participants as experts**

Individuals are being invited to participate in focus groups because they are perceived to possess important knowledge regarding particular experiences, needs, or perspectives, we hope to learn more about as a result of the needs assessment. Let participants know that you are there to learn from them and help them understand the importance of their contribution.

- **Your role as moderator /facilitator**

It is important to present yourself as a **moderator /facilitator** rather than a friend. You will need to let participants know that you are part of a team that is conducting a study for the needs’ assessment of a particular scientific group. This formality will indicate to the participants that their participation is important and will contribute to the needs’ assessment.

- **Balancing rapport and professionalism**

Part of your role is to achieve a balance between building rapport with participants and conveying an appropriate level of professionalism. Your role during the focus groups is not that of a good conversationalist or a friend who provides feedback, but of a professional. If you are too casual, participants may not see you as someone who is prepared to take what they have to say seriously. However, if you are too formal, participants may feel intimidated by you and may not be as willing to reveal information. Strive to achieve a balance between being formal and casual during your focus groups.

- **Recognizing and appreciating participants for their time and contribution**

This is one of the most important things you can do to help create rapport. Remember to thank participants for their time and participation. Let them know that the information they have shared is valuable for the project.

LISTENING SKILLS

- **Listen carefully to the participants**

Active listening allows you to probe effectively and at appropriate points during the focus group. Active listening involves not only hearing what someone is saying, but also noticing body posture and facial gestures (i.e., any changes in nonverbal behaviour) that might provide clues as to the appropriate or necessary ways to engage participants.

- **Show participants you are listening**

Show participants that you are listening to what they are saying. Signs that you are paying attention may include leaning forward slightly, looking directly at participants while they are speaking, or nodding at appropriate times. Such behaviours not only indicate that you, as the facilitator, are more engaged, but also will help maintain participants’ engagement. Looking away, yawning, or frequently checking your watch will most likely make participants feel that you are not paying attention. If the participants suspect that you are not listening to them with interest, they may take their role of sharing expert knowledge less seriously and, therefore, may not elaborate or provide much detail with their answers.

- **The importance of neutrality during the interview**

While showing participants that you are actively listening and interested in what they are sharing, you will also want to remain as neutral or impartial as possible, even if you have a strong opinion about something. Use phrases such as “Thank you. That is helpful.” Comments such as “I can’t believe it!” or “You really think that?!” are not appropriate remarks for a facilitator to make, because they infer your opinion and impose judgment on the participant, which will probably shut down the discussion.

QUALITIES OF AN EFFECTIVE FOCUS GROUP MODERATOR/FACILITATOR

**Roles and Responsibilities of moderators/facilitators:**

- Keep participants focused, engaged, attentive and interested
- Monitor time and use limited time effectively
- Use prompts and probes to stimulate discussion
- Use the focus group guide effectively to ensure all topics are covered
- Politely and diplomatically enforce ground rules:
  - Make sure everyone participates and at a level that is comfortable
  - Limit side conversations
  - Encourage one person to speak at a time
- Be prepared to explain or restate questions
- Diffuse and pre-empt arguments
- After the focus group, work with the note taker to discuss the uprising themes. To facilitate the debriefing discussion, review the notes of the discussion, discussing areas that seemed particularly important or salient given your knowledge of the research questions. [Need to determine who will take responsibility for these notes, as well as the consent forms, and tapes of the focus group discussion.]

**Effective moderators:**

- Have good listening skills
- Have good observation skills
- Have good speaking skills
- Can foster an open and honest dialogue among diverse groups and individuals
- Can remain impartial (i.e., do not give their opinion about topics, because this can influence other people’s perspectives)
- Can encourage participation when someone is reluctant to speak up
- Can manage participants who dominate the conversation
- Are sensitive to gender and cultural issues
- Are sensitive about the differences in power among and within groups

**Roles and Responsibilities of Note Takers:**

- Bring the following materials for the focus group:
- Materials to record the focus group, including writing utensils (more than one, in case a pencil breaks or a pen runs out of ink) and a lot of paper
- Bring a flip chart as well as markers of different colors for recording information (as needed) on a flip chart or dry erase board. NOTE: if a dry erase board is used instead of a flip chart, be sure that dry erase markers are available or that you bring this type of marker.
- Tape for affixing flip chart pages on the wall, as needed.
- Recording equipment: a tape recorder, extension cord, extra tapes and extra batteries
- Ensure that ground rules for the focus group are written clearly and neatly on a flip chart (it may be helpful to do this beforehand)
- Assist the facilitator in arranging the room (e.g., seating, flip chart stand and paper, placement of the ground rules, etc.)
- Record major themes, ideas, comments and observations regarding group dynamics in hand-written notes
- Conduct a debriefing discussion with the focus group facilitator immediately after each focus group. To facilitate the debriefing discussion, review your notes with the focus group facilitator. Capture any new insights that emerged as a result of this discussion with the facilitator.
- Do not throw away any papers with notes of the focus group discussion. These will be stored with other data collected through the needs’ assessment.
- [Need to determine who will take responsibility for these notes, as well as the consent forms, Debriefing Discussion Tool and tapes of the focus group discussion.]

**Effective Note Takers:**

- Have good listening skills
- Have good observation skills
- Have good writing skills
- Can take notes that are comprehensive but not word-for-word
- Act as an observer, not as a participant
- Can remain impartial (i.e., do not give her/his opinions about topics, because this can influence what people say)

TIME MANAGEMENT

- **Managing time during the interview**

Individuals love to talk about their experiences and may tend to go on and on about them. Here is where your skills as an interviewer are put to the test. As the interviewer, your job is to structure the interview in such a way that you elicit a complete response to questions, probing insightfully so that you get the level of detail you need to arrange the issues adequately.

- **Keep the interview moving**

It is also your responsibility to politely lead the interview forward when what the respondent is sharing is less useful given your topics of discussion. Other times, you may want to acknowledge that your time together is waning and there are some other aspects of their work and experience that you want to be sure you have time to learn about and explore, and, for this reason, you are going to move on

## Supplementary Materials 5

Key findings from Desktop Literature Review and Qualitative research (FGs discussions and PIs)

|  | | | **Desktop Literature review** | **Qualitative Research** |
| --- | --- | --- | --- | --- |
| **Entitlement** | Legal | *Barriers* | - Host countries may lack policies and other legal documents with specific recommendations on immunisation for refugees and other migrants  - In host countries, migrants may not be specially included in National Immunisation Programs (NIPs)  - Migrants may have differences in entitlement to health and immunisation services when they arrive in host countries either with respect to the local population and among different groups (e.g. asylum seekers, refugees, undocumented migrants)  - Health policies towards refugees and other migrants vary significantly among host countries and may influence their access to vaccination across countries. | -Focus Groups and Interviews showed that in all countries, there is an absence of protocols specific to the immunization of NAMs. Procedures seem to be much more explicit concerning asylum seekers, however, public policy institutions have not considered rights to vaccination specifically for NAMs.  - Participants in Spain emphasised the importance of universal healthcare for all.  - Even though Germany, Greece and have free access to vaccinations, NAMs from Third countries (not including the EU) are not prioritized mainly due to a lack of availability of vaccines/ vaccine flow. |
|  |  | *Solutions* | - Legal status should not be taken into account for the decision on access to vaccinations.  - Avoid relying on regularisation of legal status because it would unacceptably delay the protective effects of in particular for high-risk population groups (unaccompanied minors, children, pregnant women and the elderly) | - In Greece, a prioritization is provided for vaccines against diseases that can cause outbreaks / epidemics, i.e. MMR. - In Cyprus, NAMs from Third countries receive standard vaccinations in reception camps (polio, tetanus and diphtheria), but this process is characterized by long delays. |
|  | Economic | *Barriers* | - In some host countries there is a lack of clarity on whether there is a cost/fee for people who are not enrolled in national health system/ insurance plans - Many migrant groups do not have access to free health care and vaccines upon arrival in some host countries. | Malta stresses a financial barrier concerning the immunization of Third country nationals who apply for work permits, since vaccinations are needed when applying for a job. Work permit seekers are urged to pay for vaccinations themselves |
|  |  | *Solutions* | None from literature | Nothing relevant among key findings from qualitative research |
| **Reachability** | Organizational | *Barriers* | - The lack of identification documents is a significant obstacle for reaching migrants. Many refugees and migrants, through fears of legal problems, may have chosen not to keep, or have lost, any personal documentation including vaccination records. - Difficulty of identifying migrants from health registries because they often lack registration with the NHS or health insurance - Difficulties in receiving and understanding invitation letter: even if migrant groups are on the register they have a high mobility and may not understand the invitation letters sent to attend for vaccination - Lack of procedure to reach migrants at community level. Only half of the EU countries had policies and procedures to ensure migrants’ access to vaccination at the community level, while all have it only at point of entry - Difficulties with NGOs, which often support communication and access for refugees and asylum seekers, in providing ongoing care and to coordinate with other organisations | - Locating NAMs to organize immunizations seems to be a challenging procedure for all countries, since there is lack of well-organized immunization records/data bases for the specific population.  - The main challenges concerning vaccination are when NAMs start to live in the wider community: ● They often move to another country (this is especially true for first entry countries) ● They often change address or move to another region without informing about their new address hence invitation letters for booster shots (where available) cannot be delivered. ● Thy often change contact mobile numbers hence messages concerning up-coming immunizations, immunization certificates cannot be sent to them. ● Undocumented NAMs avoid visiting vaccination centers, due to fear of deportation.  ● Lack of culturally sensitive and language specific campaigns for NAMs about immunizations |
|  |  | *Solutions* | - Flexible solutions to overcome problem of identification -Periodic Intensification of Routine Immunization (PIRI) services in target areas could be a potentially important intervention and a useful approach for reaching marginalized people who may remain underserved by routine service delivery and primary health care  -To facilitate community access and proximity health options, it is necessary to identify different vaccination solutions according to specific situations, always giving priority to the proximity of public health options -For migrant reception centres, possible options are: ●in the same reception centre (preferably for large centres) through existing services or through the use of mobile units or  ●at vaccination centres (in the case of small reception centres), possibly including accompanying services and linguistic and cultural mediation. -Utilise mobile clinic/team as vaccination centre -Networking and coordination with NGOs. There is a need for improvement in communication with asylum seekers and coordination between agencies within and beyond the medical system. - Reach high-risk groups through advocacy, communications and supplementary immunization, including door-to-door initiatives, such as checking of immunization cards, distribution of information materials (in native languages) and vaccination campaigns | NAMs for immunizations depends on: ● Migrant status: asylum seekers can be easily reached in reception centers, when first entering the country. ● Age of NAMs: children can be reached through schools, as certain vaccinations are mandatory/encouraged when enrolling in schools. ● Gender: female NAMs can be reached and informed about vaccinations (infant, child and adult vaccination) through gynecological/maternity visits and reproductive clinics/ programs. - There is need for development of proximity strategies based on best practices in each country: ● Germany: need to develop closer collaboration among public policy officials and NGOs ● Greece: information and sensitization of the particular population about vaccinations is an important milestone towards their compliance, and the continuation of vaccination on their own free will. ● Italy: regarding proximity strategies, it is important to focus on the construction of “vaccination pathways” and not only on the provision of single services. Moreover, all sectors of the health system (even administration and registration) that come into contact with NAMs, should be able to promote and encourage immunization. Vaccination mandates could also be extended beyond schools, to other organizations such as the police, labor offices etc. that could require evidence of vaccinations. ● Cyprus: the involvement of health visitors and nursing personnel in informing NAMs about immunization after receiving training on cultural competence. ● Poland: adequate, culturally competent and easily accessible information provided to NAMs about vaccinations i.e. translated leaflets, materials, and information sessions. |
| **Adherence** | Legal | *Barriers* | Reporting undocumented migrants to immigration authorities and, more in general policies of other government sectors (such as immigration, justice or interior and home affairs) on deportation of irregular migrants also influence utilisation of vaccination services by refugee and migrant groups | Nothing relevant among key findings from qualitative research |
|  |  | *Solutions* | - Setting up vaccination centres that do not require formal identification - Coordination among governmental agencies from health and immigration sectors will be needed to ensure vaccination programs are not used by immigration authorities for tracking or enforcement | Nothing relevant among key findings from qualitative research |
|  | Economic | *barriers* | - In some host countries, migrant groups have experienced delays in access to care, and therefore low vaccination rates, due to inability to pay for care and vaccines. - Direct health costs and additional external costs associated with seeking care can dissuade migrants from asking for vaccination | Nothing relevant among key findings from qualitative research |
|  |  | *Solutions* | Health system should ensure that migrants and refugees benefit from easy access to the vaccines offered free of charge under the national vaccination schedule. The integration of free or affordable vaccination services would be more effective in increasing the participation of hard-to-reach communities. | Nothing relevant among key findings from qualitative research |
|  | Organizational | *Barriers* | - Health professionals may lack awareness and experience in providing (correct) advice on health care and vaccination for refugees and migrants - Decentralised health systems and lack of coordination among local authorities and international stakeholders may have disproportionate negative effects on refugees and migrants; - In some host countries (i.e. UK) migrants groups face barriers to access to primary care and vaccinations due to lack of access points; - Transportation issues are observed to be other barriers to immunisation - Registration through dedicated (online) systems are often required prior to vaccination, which can be confusing, and which often also imply other barriers (technological requirements, language barriers, fear of tracking tools that may lead to arrest or deportation) | Nothing relevant among key findings from qualitative research |
|  |  | *Solutions* | - Organisational solutions proposed are focused on  ● tailoring immunisation services  ● strengthening communication and social mobilisation toward specific population targets ● developing proximity approaches and the use of bus as mobile clinic buses ● actively engaging with affected communities - Strengthen collaborations with local government, relevant charities and community groups, civil society groups, social care services, public health teams and health professionals to develop engagement strategies with migrant communities. | - Developing synergies between NGOs active in the field and public organizations responsible for NAMs vaccination, i.e. in Greece information sessions are usually organized by NGOs active in the field, with the assistance and cooperation of the social services of camps/ reception centers that are more familiar with beneficiaries and can inform them accordingly; in Germany NGOs are linked to a specific contact person at state level, and therefore vaccination efforts of migrants run smoothly. - Organizing and implementing information sessions with community leaders about the importance of specific vaccines. It seems that community leaders have an important impact on beneficiaries and affect adherence (vs hesitancy) to vaccination.  - Use of mobile units or “mobile vaccination busses” - Progressively involve all members of a household in vaccinations. Starting usually from the child reach out to other members so progressively involving all the family members in vaccinations. - Providing assistance / support during actual vaccination ● For vaccinations taking place in camps (e.g. mobile units or vaccines performed by medical services inside camps), health professionals suggest the following best practices in combination or individually: (a) door-to-door visits in order to inform beneficiaries about the upcoming vaccination, (b) a note on their door about the day and time of the event, (c) sending mobile messages (sms) to all beneficiaries who have a mobile phone, (d) posting on social media (camp page, community leaders). All this information needs to be translated into migrant languages.  ● Based on existing vaccination records that often organizations seem to keep (camps, NGOs, national public services): (a) informing beneficiaries about vaccinations and booster vaccinations –letters need to be translated in the NAMs languages, (b) making appointments, if needed, (c) escorting and interpreting, if required. - Vaccinations need to be promoted by all services caring for migrants. A particular quote emphasizes this. A participant says for example when patients visit a facility for an issue irrelevant to vaccination an information leaflet or brochure can be provided regardless of the fact that vaccination was not the reason for visiting. - Linking vaccinations to employment, as reflected with in the COVID experience and the “green pass”. - Another trigger for vaccination, for those migrants who aim at continuing their journey to other European countries, is making vaccination compulsory in order to be able to continue their journey |
|  | Psycho-social | *Barriers* | - Lack of motivation and the low risk perception: immunisation is something they do not usually go through in their minds due to competing priorities (new arrivals need to balance finding employment and housing, alongside getting catch-up vaccines) - There is a stigma around some infectious diseases and fear of accessing care due to precarious immigration status. Fear of being asked of their legal status or being identified were always present, affecting the everyday life of the migrant families and children with no legal documentation - Mistrust in the health and vaccination system. Some migrant groups (e.g families, workers) may have well-founded mistrust of government and health organisations (based on previous experiences). This lack of trust in the health system causes reluctance to consent | Nothing relevant among key findings from qualitative research |
|  |  | *Solutions* | - Ensure all that vaccination is voluntary, confidential, non-stigmatizing - Communication campaigns should be organized to clarify the benefits of vaccination and the total lack of legal consequences in order to avoid the fear of repatriation or removal - Organize vaccination through trusted community health workers or non-profit organisations to avoid fear of legal consequences | In order to overcome these barriers participants emphasize the need for building relationships of trust by using mediators for example as well as community leaders who can boost vaccination uptake. These strong social figures can support |
|  | cultural-linguistic | *Barriers* | - Migrants may lack awareness of vaccines’ existence. The lack of knowledge of the health system, vaccination schedule and relevant VPDs in the host country are important barrier to vaccination uptake - Migrants may have misinformation and beliefs about infectious diseases and their risk and transmission factors that hinder the acceptability of screening and vaccination. - Belief that vaccine is unnecessary and optional. Traditional beliefs of migrants may play a role in the value placed on outcomes of infectious disease interventions - Concern about side effects and safety. Some parents are mainly concerned that their children would develop high fever after immunisation and needing medical attention. Others may also worry about the pain associated with the injection. - Several barriers about the lack of cultural competence of the services are reported in literature - The lack of appropriate language aids is a major barrier for access to the health system by refugees and migrants. Poor information resources in their native language can reduce confidence and ability to access vaccination, as well as confidence and adherence to recommendations - Professionals or administrative personnel who are not adequately and culturally trained (they are not only likely to contribute to a lower quality of service, but also may assume detrimental inappropriate behaviours and stereotypical attitudes towards migrants) | Nothing relevant among key findings from qualitative research |
|  |  | *Solutions* | - Health promotion and educational programs are especially important for refugees and migrants because often they are not familiar with the health and vaccination systems of the host country and so lack knowledge of what kind of support is available, whether they are entitled to it and how they can access it  - Specific subgroups of migrants should be taken into account in the implementation of the general measures proposed to overcome linguistic barriers, which include the use of information material translated into different languages, staff training and the activation of services tailored to the specific needs of migrants and the identification and training of foreign cultural mediators to inform and motivate migrants on access to vaccination services - Information regarding immunisation and informed consent should be available in multiple languages - Policy-makers must guarantee a culturally competent healthcare service? - The cultural competence and cultural awareness of healthcare practitioners and vaccinators need to be strengthened to adequately respond to the needs of refugees and migrants: the more awareness among practitioners about the cultural background of their patient, the higher the quality of care they can provide. - Generating and maintaining demand for immunisation services and addressing vaccine hesitancy will require use of traditional and new social communication platforms, optimising the role of front-line health care workers, identifying and leveraging immunisation champions and agents of change, tailoring immunisation programme advocacy and communication to susceptible populations, including mobile, marginalised and migrant populations, and communicating the benefits of immunisation and the risks presented by VPDs - Communication and advocacy strategies regarding the benefits and safety of vaccination, including engagement of mainstream and social media and other relevant channels, should be tailored to ensure that evidence and information reach target refugee and migrant communities. - Direct involvement of immigrant communities and cultural mediators and / or third sector associations to encourage the transmission of key messages for prevention in the languages understood by migrants and in a culturally appropriate manner and to prevent the dissemination of incorrect information  - The use of cultural mediators can be helpful in facilitating productive cross-cultural patient–provider dialogue and should be encouraged within immunisation programmes. Such mediators have been found to be effective educators, health promoters and health-care system navigators for refugees and migrants, mitigating key barriers to care. | Training on how (a) to communicate the need for vaccinations to migrants as well as (b) how to discuss fears, misunderstandings or misinformation. |
| **Achievement** | Organizational | *Barriers* | - Specific documents are often required for vaccination, creating a spectrum of barriers that IOM graduates from low to high;  ● low: some countries will accept any form of ID, valid or not, expired or not, and from anywhere, only to verify the identity;  ● medium: other countries require specific types of documents (e.g. residence permit, host country insurance cards), which constitutes a higher barrier, but those documents are accepted even if expired;  ● high: other countries require specific types of documents that are still valid. - Unknown vaccination history and lack of vaccination records - Many vaccines require booster doses and timed intervals of weeks or even months to get full immunisation. Ensuring appropriate follow-up and the completion of vaccination schedule within and across countries is difficult when dealing with migrants. - Lack of recording of vaccination doses and information is not shared with risk of duplication of vaccination doses - Vaccine price and limited supply doses may affect particularly marginalised communities  - Health system capacity: lack of financial and human resources - Fragmented health systems and diverse models of care, with separate pathways for screening and vaccination of refugees and migrants, can create confusion for both patients and health-care providers especially when lead with large influx of refugees and migrants - Organization and opening of services is inconvenient, considering the need of multiple appointments; lack of time for dedicated outpatient visits. | Key findings for execution and completion do not differ to those presented for adherence and hesitancy. |
|  |  | *Solutions* | - Administrative flexibility to facilitate the access to vaccination to those who have no registration with the NHS or valid documents has been adopted in several countries for COVID-19 - If the previous vaccination documentation is difficult to interpret because of language barriers, and there may be doubts about its authenticity, it may be preferable to ignore the written record and repeat the vaccination.  - If previous immunisation is not available, immigrants should be considered susceptible to the disease in question, triggering the age-appropriate vaccination schedule - The reception centres are an ideal setting to offer full or catch-up vaccination. The operational mechanism for vaccination should essentially be time accommodation dependent, which may require a minimum of 6-7 months. For migrants who may move across borders in their migration, high-quality data need to be collected and shared between countries to facilitate completion of vaccination doses. - Strengthening partnerships and implementing initiatives across countries of arrival, transit and destination to develop and share better documentation in order to ensure immunisation and avoid revaccination - Innovative Solutions and tailored approaches - The importance of a coordinated response to ensure vaccination provision is prioritised in situations of sudden large influxes of refugees and migrants. Intersectoral stakeholders should be engaged in the development of action plans to improve country preparedness, and public–private partnerships might be used to address issues of vaccine supply to cater for sudden influxes of refugees and migrants. Additional financial and human resources should be available for appropriate service delivery strategies - A single-dose vaccine option may be preferable for (e.g. seasonal workers) who move between locations/states - Extended clinical hours, out-of–hours clinics, drop-in centres and pharmacy-based delivery of vaccination can provide opportunities for administering vaccination and reduce socioeconomic barriers to accessing care (within immunisation service delivery regulations and laws in the country) - The Health Service Executive’s active recall system for the primary immunisation programme. Clinics for the school immunisation programme held over the summer for those children who may have missed vaccination day. | Key findings for execution and completion do not differ to those presented for adherence and hesitancy. |
|  | cultural-linguistic | *Barriers* | - Language barriers and lack of interpreters for informed consent - Insufficient training among Health Professionals - Staff shortages, including for cultural mediators and interpreters, who are critical for establishing effective and inclusive services, act as barriers to implementation of national immunisation policies and limit systematic collection and evaluation of data for corrective actions | Key findings for execution and completion do not differ to those presented for adherence and hesitancy. |
|  |  | *Solutions* | - Communication services (i.e. cultural mediators, interpreters) for health care providers should be available, or improved, for the promotion of an inclusive and culturally sensitive health system - It is necessary to provide training opportunities for Health Professionals to improve their awareness of the catch-up needs of refugees across all age groups. Resources such as online immunisation calculators, refugee specific guidelines and e-learning could potentially equip Health Professionals with the relevant skills and knowledge and ultimately make implementation of catch-up vaccines for this group easier. - Train immunisation managers and service providers. | Key findings for execution and completion do not differ to those presented for adherence and hesitancy. |
| **Evaluation** | Organizational | *Barriers* | Lack of robust and standardised data Information on the effectiveness and cost-effectiveness of vaccination programmes targeting migrants, and the practical implementation challenges facing these interventions | - Across all countries, there is lack of national, regional or local data bases to record vaccinations as a way of monitoring vaccination schedules and avoiding unnecessary vaccinations or missing booster vaccinations (for Greece, Malta and Poland the specific barrier was also mentioned in the section about reachability of NAMs) - In all countries apart from Germany, different actors involved in the immunization of NAMs seem to keep their own records and data, however they use them internally. |
|  |  | *Solutions* | - Track each individual’s immunisation status, preferably through introduction of electronic immunisation registries that are well integrated within health information systems and leverage other relevant civil registries. - Electronic medical records, interlinking national immunisation registers and data sharing along migratory routes can contribute to monitoring and planning of vaccination of refugees and migrants.  - Setting up or expanding immunisation information systems to monitor vaccination coverage - Further research would be valuable in order to better understand and evaluate acceptability and accessibility of migrant communities toward interventions - Immunisation information systems, population-based immunisation registries, record administered vaccinations to support immunisation decision-making at the local level and to guide policies and programmes for public health operations. | - The need for improving immunization data registration is emphasized by all participants. - Moreover, there is a need for data collection, in order to evaluate relevant initiatives and promote best practices –advocate them to policy makers, and disseminate them to different actors. - In Germany a collaboration seems to be in effect among different bodies in terms of monitoring vaccination status of NAMs, schedules as well as advocate and disseminate best practices. |
